# Sub-okra leaf shape conferred via chromosomal introgression from *Gossypium barbadense* L. improves photosynthetic productivity in short-season cotton (*Gossypium hirsutum* L.)

**DOI:** 10.3389/fpls.2024.1393396

**Published:** 2024-07-18

**Authors:** Hui Jiang, Xiongfeng Ma, Jialiang Shi, Mingwei Gao, Xianliang Zhang, Chao Zhang, Qichao Chai, Yongcui Wang, Xiuli Wang, Jiabao Wang, Ying Chen, Donglou Zhang, Fengrui Li, Wenchao Zhao, Junsheng Zhao

**Affiliations:** ^1^ Institute of Industrial Crops, Shandong Academy of Agricultural Sciences, Jinan, Shandong, China; ^2^ Institute of Cotton Research of Chinese Academy of Agricultural Sciences, Anyang, Henan, China; ^3^ Institute of Cotton Research of Dezhou Academy of Agricultural Sciences, Dezhou, Shandong, China

**Keywords:** photosynthesis, canopy structure, introgression segment, sub-okra leaf shape, short-season cotton

## Abstract

Leaf shape is a vital agronomic trait that affects plant and canopy architecture, yield, and other production attributes of upland cotton. Compared with normal leaves, lobed leaves have potential advantages in improving canopy structure and increasing cotton yield. A chromosomal introgression segment from *Gossypium barbadense* L. conferring sub-okra leaf shape to *Gossypium hirsutum* L. was identified on chromosome D01. To determine the effects of this transferred sub-okra leaf shape on the leaf anatomical characteristics, photosynthesis-related traits, and yield of short-season cotton, we performed a field experiment with three sets of near-isogenic lines carrying okra, sub-okra, and normal leaf shape in Lu54 (L54) and Shizao 2 (SZ2) backgrounds. Compared with normal leaves, sub-okra leaves exhibited reduced leaf thickness and smaller leaf mass per area; moreover, the deeper lobes of sub-okra leaves improved the plant canopy structure by decreasing leaf area index by 11.24%–22.84%. Similarly, the intercepted PAR rate of lines with sub-okra leaf shape was also reduced. The chlorophyll content of sub-okra leaves was lower than that of okra and normal leaf shapes; however, the net photosynthetic rate of sub-okra leaves was 8.17%–29.81% higher than that of other leaf shapes at most growth stages. Although the biomass of lines with sub-okra leaf shape was less than that of lines with normal leaves, the average first harvest yield and total yield of lines with the sub-okra leaf shape increased by 6.36% and 5.72%, respectively, compared with those with normal leaves. Thus, improvements in the canopy structure and photosynthetic and physiological characteristics contributed to optimizing the light environment, thereby increasing the yield of lines with sub-okra leaf shape. Our results suggest that the sub-okra leaf trait from *G. barbadense* L. may have practical applications for cultivating short-season varieties with high photosynthetic efficiency, and improving yield, which will be advantageous for short-season varieties.

## 1 Introduction

Leaves play a critical role in light capture during photosynthesis, gas exchange, and water transport in crops (Tsukaya, 2006). In particular, leaf shape is a key aspect related to the plant type and canopy structure, and it affects light interception by cotton crops (Du et al., 2009; Feng et al., 2016; Chen et al., 2021; Wang et al., 2021). *L-D_1_* is the main locus regulating the development of four major leaf shapes in upland cotton: super okra (*L_2_s*), okra (*L_2_o*), sub-okra (*L_2_u, L_2_e*), and normal (*l_2_*) (Jiang et al., 2015). Map-based cloning showed that the *L-D_1_* locus was similar to the *Late Meristem Identity* 1 (*LMI1*) gene. Furthermore, virus-induced silencing of this gene in an okra leaf-shaped variety was sufficient to recover normal leaf formation (Chang et al., 2016; Andres et al., 2017). Sub-okra shape is the ancestral leaf shape of tetraploid cotton that gave rise to the okra allele, and the normal shape is derived from a mutant allele that came to predominate and define the leaf shape of cultivated cotton; however, the relationship between *L_2_u* and *L_2_e* is still unclear (Andres et al., 2017).

The leaf shape of most upland cotton varieties has been designated as normal. The introduction of other leaf shapes with variable indentation depths of the leaf blades has been associated with production advantages, such as accelerated flowering rate, early maturity, reduced boll rot and lint trash incidence, increased whitefly and pink bollworm resistance, and higher foliar chemical application efficacy (Jiang et al., 2015). However, different studies have obtained inconsistent results on the effects of leaf shape on insect resistance, yield, photosynthetic rate, and water-use efficiency (Wilson, 1990; Pettigrew et al., 1993; Jiang et al., 2015; Andres et al., 2016).

Over the past 60 years, cotton production in China has developed rapidly owing to improvements in cultivars and farming technology (Dai and Dong, 2014; Yu et al., 2016). A high-yielding cultivation system has been established in the Yellow River Valley, which is one of the major cotton-planting areas in China (Dong et al., 2006, Dong et al., 2009). In this system, full-season cotton planting requires considerable material and labor inputs, which has decreased the sustainability of this planting mode in Yellow River Valley (Dai et al., 2017). Based on short-season cotton, a new alternative has been established to reduce labor and costs (Dong et al., 2010; Lu et al., 2017; Qi et al., 2021). Short-season cotton also plays a very important role in double-cropping systems, such as garlic–cotton, rape–cotton, and wheat–cotton systems, which have been widely adopted to increase land productivity and profitability in regions with relatively abundant water resources and near-optimum temperature conditions (Qi et al., 2018). Compared with full-season cotton, short-season cotton is characterized by a shorter growth and development period, compact growth form, and relatively concentrated flowering and boll-setting; thus, it can be sown late and still mature early (Yu et al., 2005). However, the shortened growth cycle places great demands on the plant type, maturity, and yield potential of newly developed cultivars (Qi et al., 2018).

Under high planting density, a normal leaf shape and compact growth pattern might negatively affect the canopy structure and compromise the potential improvements in fiber yield of short-season cotton. Our previous study showed that the sub-okra leaf shape improved the canopy structure and photosynthetic and physiological characteristics, thereby increasing the total biomass and yield of full-season cultivars (Jiang et al., 2023). Thus, we sought to verify whether sub-okra leaf shape might also confer its advantages to short-season cotton and increase the fiber yield.

Therefore, the main objective of this study was to determine the genetic origin of the sub-okra leaf shape and the effects of leaf shape under the control of the *L-D_1_* alleles on leaf anatomical characteristics, canopy structure, yield, and fiber quality in short-season cotton. Genomic differences of near-isogenic lines were analyzed to the identification of the introgressive segment using genome resequencing technology. Physiological traits such as the leaf area index (LAI), intercepted PAR rate (IPR), relative chlorophyll content (SPAD value), leaf net photosynthetic rate (Pn), and biomass of each organ were investigated to determine the biological process by which leaf shape affects short-season cotton yield. This study is expected to provide useful information and germplasm resources for the development of short-season cotton varieties with high photosynthetic efficiency.

## 2 Materials and methods

### 2.1 Study site

The present study was conducted at the experimental station of the Dezhou Academy of Agricultural Sciences in Dezhou city (37°36′N; 116°35′E), Shandong, China. Chemical analysis of the soil samples prior to planting in 2018 indicated that the soil was sandy loam and included 13.48 g·kg^−1^ organic matter, 40.9 mg·kg^−1^ available nitrogen (N), 16.4 mg·kg^−1^ available phosphorous (P), and 98.68 mg·kg^−1^ available potassium (K). The climate and environmental conditions are shown in **Supplementary Figure S1**.

### 2.2 Plant materials and experiment design

With a genetic background of L28 NORMAL, which was a commercial upland cotton (*Gossypium hisutum* L.) variety in the Yellow River basin of China, L28 SUBOKRA carried an introgressive segment from *Gossypium barbadense* L. The plant materials used in the field experiment were near-isogenic lines with *L-D_1_* alleles in a Lu54 (L54, early maturing variety with the growth period of 106 days) and Shizao2 (SZ2, late maturing variety with the growth period of 111 days) genetic background. Both lines with normal leaf shape (**Supplementary Figure S2A**), i.e., L54 NORMAL and SZ2 NORMAL, were well-adapted commercial short-season varieties grown in the cotton region of China in the Yellow River Basin. The okra leaf shape, which is characteristic of L54 OKRA and SZ2 OKRA, was introduced from T586 (**Supplementary Figure S2B**), and the sub-okra leaf shape of L54 SUBOKRA and SZ2 SUBOKRA was introduced from L28 SUBOKRA (**Supplementary Figure S2C**). Two donor parents, T586 and L28 SUBOKRA, were backcrossed with L54 NORMAL and SZ2 NORMAL, respectively, for eight generations. The near-isogenic lines differed only in leaf shape within the same background.

Lines with okra, sub-okra, and normal leaf shapes were planted on 25 May 2018, 27 May 2020, and 25 May 2021, in plots with six rows spaced 76 cm apart. The length of each row was 8 m, and each row was thinned to 7 plants·m^−1^ for a population density of 90,000 plants·ha^−1^. For each genetic background, three lines with different leaf shapes were arranged in a randomized complete block design, with three replicates. Field management procedures, including fertilizer application, plant pruning, pest control, and chemical control, were performed according to local practices unless otherwise indicated.

### 2.3 Data collection

Physiological parameters, canopy structure, cotton lint yield, and fiber quality data were collected in 2018 and 2020. The physiological and anatomical data of sub-okra and normal leaves in different canopy layers were measured in 2021, and genome resequencing was also performed in 2021.

#### 2.3.1 Physiological measurements

Physiological parameters, including chlorophyll content, photosynthetic rate, and biomass accumulation, were determined in five plants randomly selected from each plot at 20 days, 40 days, 60 days, 80 days, and 110 days after sowing (DAS). Measurements were conducted from 9:00 to 10:00 a.m. under sunny and windless weather conditions.

For the measurement of chlorophyll content during growth, the SPAD value (relative chlorophyll content) of the fourth leaf below the main stem terminal before topping and the second from the top after topping was measured using a SPAD-502 chlorophyll meter (Konica Minolta Holdings, Inc., Tokyo, Japan). The net photosynthetic rate (Pn) of the same leaves was also measured using a Li-6800 portable photosynthesis system (Li-Cor, Lincoln, NE, USA).

To measure the biomass at different stages, randomly selected plants were manually removed from the soil. The root, stem, leaf, and fruit biomass were recorded for each sampled plant after oven drying at 108°C for 30 min, and then, at 80°C to a constant mass was reached.

For the measurement of leaf mass per area (LMA) at the full flowering stage, leaves at the main stem were selected from different top, middle, and bottom canopy layers, respectively. The area for each leaf was measured using a leaf area meter (Li-3100C, Li-Cor, Lincoln, NE, USA), then each measured leaf was placed in individual paper bags and dried at 108°C for 30 min and 80°C to a constant mass before weighing.

#### 2.3.2 Leaf anatomy

Main stem leaves from top, middle, and bottom canopy layers were sampled for anatomical analysis. The samples were fixed, washed, and dehydrated as previously described (Meng et al., 2021). Sections (approximately 5 μm thick) were cut with a microtome (Leica RM2235, Leica, Wetzlar, Germany), stained with 1% (w/v) safranin O (Amresco Inc., Ohic, USA) and 1% (w/v) fast green FCF (Merck & Co., Inc., Darmstadt, Germany), examined with a fluorescence microscope (Olympus BX51, Olympus Co., Tokyo, Japan), and photographed. Leaf thickness (LT), palisade tissue thickness (PT), spongy tissue thickness (ST), adaxial epidermis thickness (ADET), and abaxial epidermis thickness (ABET) were taken with ImageJ software. Cell tense ratio (CTR), spongy ratio (SR), and leaf density (LD) were calculated using the following equations (Meng et al., 2021).


CTR=(PT/LT)×100%

SR=(ST/LT)×100%

LD=LMA/LT

#### 2.3.3 Canopy structure

Data related to canopy structure were collected using an AccuPAR LP-80 (METERGroup, Inc., Pullman, USA). The LAI and transmitted PAR (TPAR) of the bottom canopy layer were measured at 40, 60, 80, and 110 DAS. The reflected PAR (RPAR) and incident PAR (IPAR) above the canopy were also recorded at the same time. Transmitted PAR rate (TPR), reflected PAR rate (RPR), and intercepted PAR rate (IPR) were calculated using the following equations (Xing et al., 2018).


TPR=TPAR/IPAR

RPR=RPAR/IPAR

IPR=1−TPR−RPR

#### 2.3.4 Lint yield, lint percentage, and fiber quality

Yield was determined based on three central rows per plot at harvest. Cotton was manually harvested on October 5 and 20. After sun drying for approximately 7 days, the seed cotton was weighed and ginned, and lint percentage and fiber quality were tested in each plot. Fiber quality was measured using a high-volume inspection system (Uster Technologies, Switzerland).

#### 2.3.5 Genome resequencing analysis

Young leaves of L28 NORMAL and L28 SUBOKRA were collected for DNA isolation. Genomic DNA was extracted using a polysaccharide and polyphenol plant genomic DNA extraction kit (Tiangen, cat: DP360). Genome resequencing was performed on an Illumina HiSeq2000™ by Beijing Novogene Technology Co., Ltd. (Beijing, China). The sequences of clean reads were mapped to the TM-1 and Hai7124 genomes (http://cotton.zju.edu.cn/).

#### 2.3.6 DNA marker detection

DNA from L28 NORMAL and L28 SUBOKRA was amplified with a T-100 PCR instrument (Bio-Rad, USA) and GoTaq^R^ DNA polymerase reaction system (Promega Beijing Biotechnology Co., Ltd., Beijing, China). Primers were synthesized by Beijing Qingke Xinye Biotechnology Co., Ltd. (Beijing, China). The sequence of primers was downloaded (http://cotton.zju.edu.cn/) and is listed in **Supplementary Table S1**. The PCR products were separated by electrophoresis on 8% (mass fraction) non-denaturing polyacrylamide gel and observed by rapid silver staining.

### 2.4 Statistical analysis

The experimental data were analyzed using the DPS data processing system (Tang and Feng, 1997). Means were separated using Duncan’s multiple range test at p = 5%. Graphs were drawn using SigmaPlot software V10.0 (Systat Software, Inc.).

## 3 Results

### 3.1 Detect of chromosome segment conferring sub-okra leaf shape

**Figure 1:**
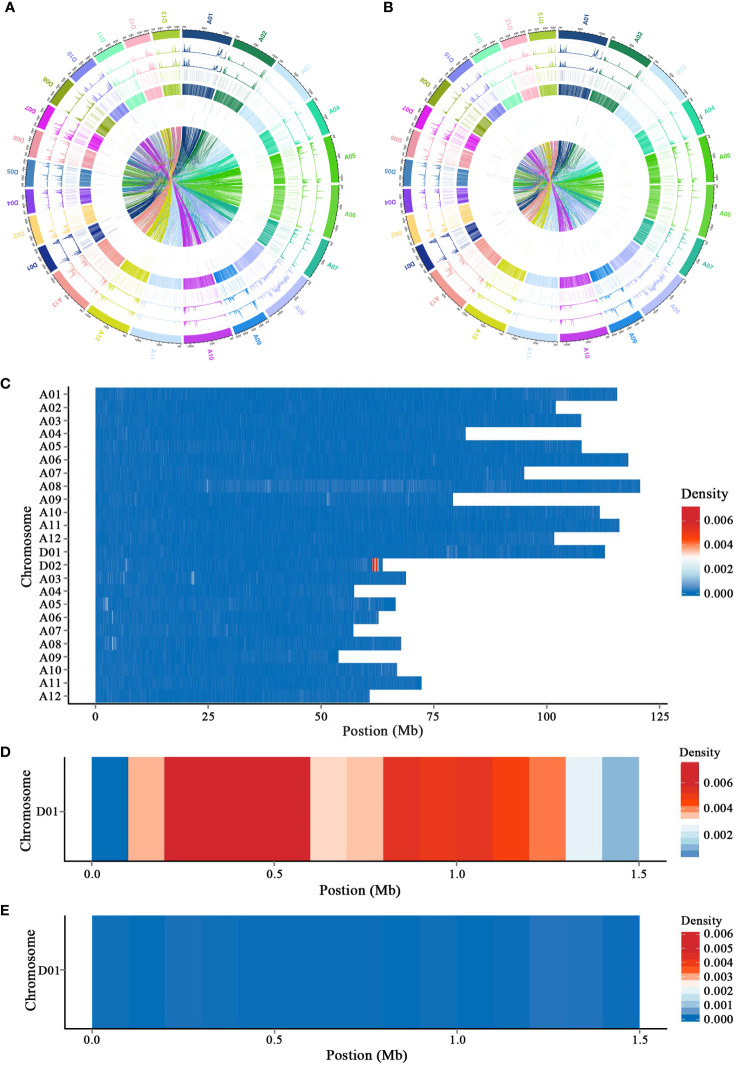
Detection of chromosomal ingression segment with genome resequencing. **(A, B)** Distribution of genomic variations in L28 NORMAL and L28 SUBOKRA compared to TM-1. **(C)** Difference in genomic variations between L28 NORMAL and L28 SUBOKRA. **(D, E)** Genomic variations of introgression segment in L28 SUBOKRA compared to TM-1 and Hai7124.

Genomes of L28 NORMAL and L28 SUBOKRA were sequenced on an Illumina HiSeq2000™. Clean data were mapped to the TM-1 and Hai7124 genomes, and the genomic variations were also detected (**Figures 1A, B**; **Supplementary Figures S3A, B**). After analyzing the genomic variations between L28 NORMAL and L28 SUBOKRA, a segment of approximately 1.5 Mb with much-enriched variations was detected on chromosome D01 of L28 SUBOKRA (**Figure 1C**). Sequence alignment with the TM-1 and Hai7124 genomes revealed that the chromosome segment came from *Gossypium barbadense* L. (**Figures 1D, E**), and 123 genes were included in this segment (**Supplementary Table S2**). A total of 20 specific indel markers were identified for the detection of this chromosomal introgression segment (**Supplementary Figure S4**).

### 3.2 Physiological characteristic of leaves at different canopy layers

**Figure 2:**
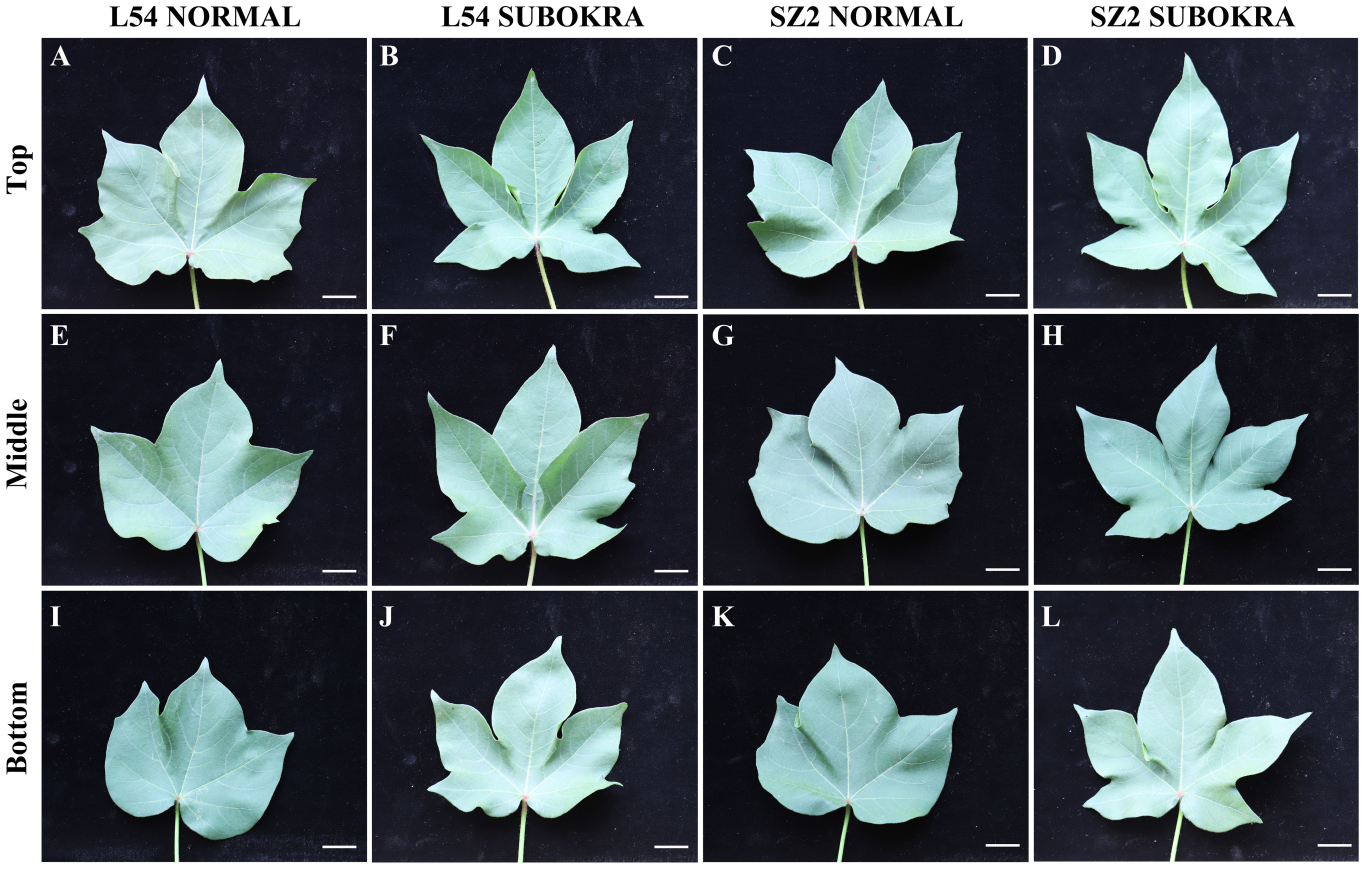
Morphology of leaves in different canopy layers of near-isogenic lines. **(A–D)** Leaves in top layer of line L54 NORMAL, L54 SUBOKRA, SZ2 NORMAL, and SZ2 SUBOKRA, respectively. **(E–H)** Leaves in middle layer of line L54 NORMAL, L54 SUBOKRA, SZ2 NORMAL, and SZ2 SUBOKRA, respectively. **(I–L)** Leaves in the bottom layer of line L54 NORMAL, L54 SUBOKRA, SZ2 NORMAL, and SZ2 SUBOKRA, respectively. Scale bar, 2 cm.

**Figure 3:**
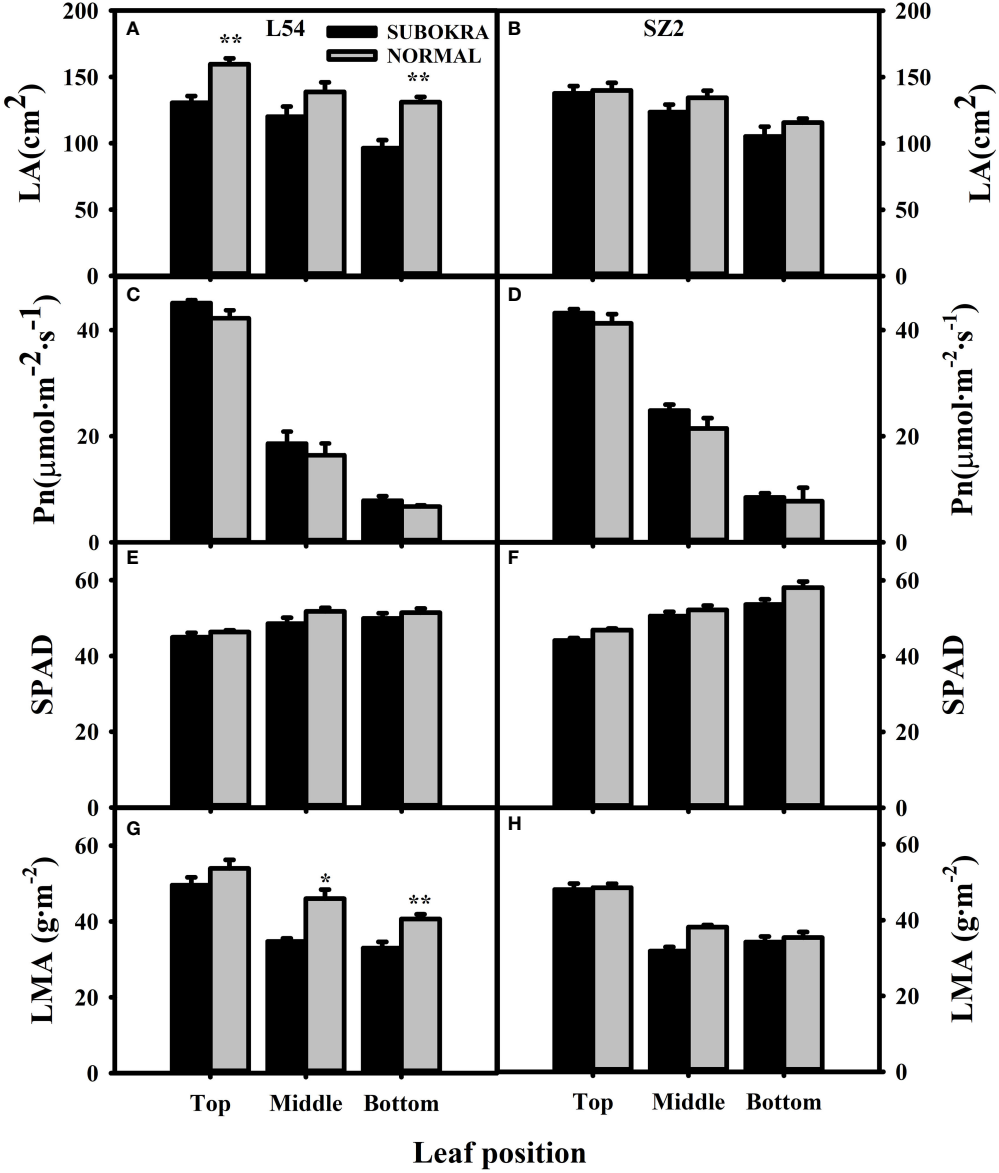
Photo-physiological characteristics of leaves in different canopy layers of near-isogenic lines. **(A, B)** LA of different layers of near-isogenic lines in genetic background of L54 and SZ2. **(C, D)** Pn of leaves in different layers of near-isogenic lines in genetic background of L54 and SZ2. **(E, F)** SPAD of leaves in different layers of near-isogenic lines in genetic background of L54 and SZ2. **(G, H)** LMA of leaves in different layers of near-isogenic lines in genetic background of L54 and SZ2. *p<0.05; **p< 0.01.

Deeper leaf lobe depth and smaller lobe width confer a slight decrease in LA to sub-okra leaves (**Figures 2A–L**, **3A, B**), which increases the PAR of different canopy layers in both genetic backgrounds (**Supplementary Figures S5A, B**). The Pn of sub-okra leaves was higher than that of normal leaves in different canopy layers (**Figures 3C, D**). Compared to normal leaves, the Pn of L54 SUBOKRA leaves was increased by 6.77%–16.69% and that of SZ2 SUBOKRA leaves was also increased by 4.65%–15.60%. The SPAD and LMA of sub-okra leaves were lower than those of normal leaves (**Figures 3E–H**). SPAD of L54 SUBOKRA and SZ2 SUBOKRA leaves was decreased by 2.94%–6.28% and 3.07%–7.74%, respectively. The LMA of L54 SUBOKRA and SZ2 SUBOKRA leaves was also decreased by 8.16%–28.37% and 1.02%–16.57%, respectively.

### 3.3 Anatomical characteristic of leaves

**Figure 4:**
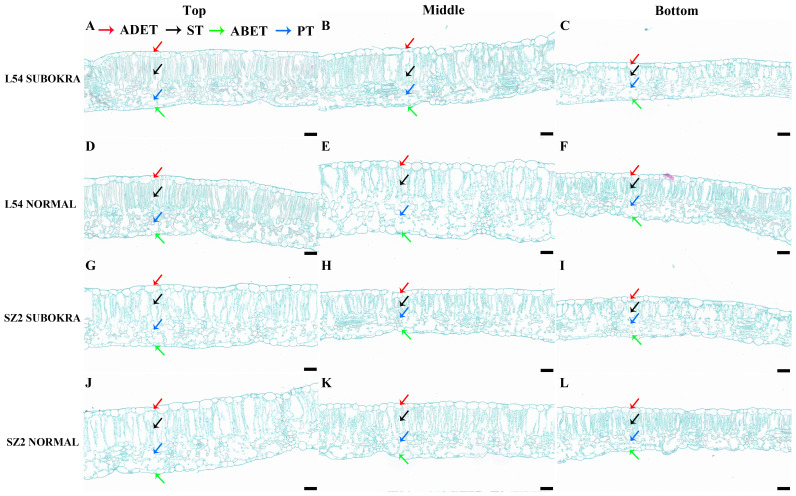
Anatomical structure of leaves in different canopy layers of near-isogenic lines. **(A–C)** Leaves in each layer of line L54 SUBOKRA. **(D–F)** Leaves in each layer of line L54 NORMAL. **(G–I)** Leaves in each layer of line SZ2 SUBOKRA. **(J–L)** Leaves in each layer of line SZ2 NORMAL. Scale bar, 50 μm.

**Figure 5:**
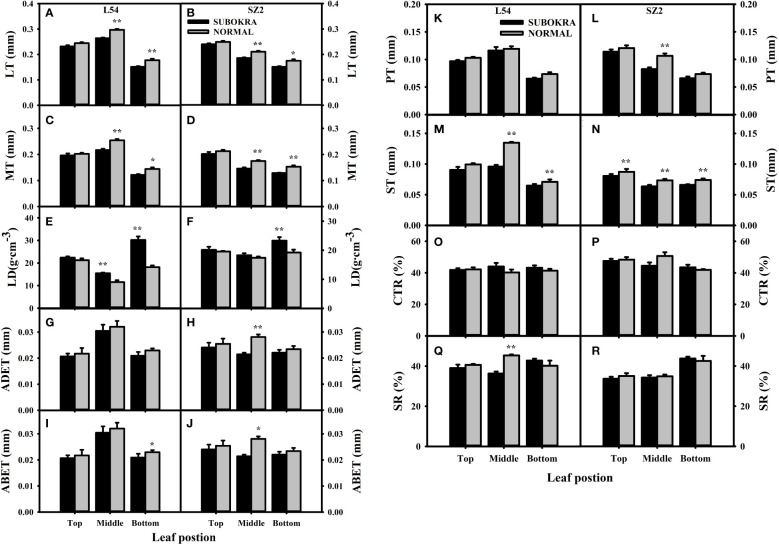
Analysis of anatomical characteristics of leaves in different canopy layers of near-isogenic lines. **(A, B)** LT of leaves in different layers of near-isogenic lines in genetic background of L54 and SZ2. **(C, D)** MT of leaves in different layers of near-isogenic lines in genetic background of L54 and SZ2. **(E, F)** LD of leaves in different layers of near-isogenic lines in genetic background of L54 and SZ2. **(G, H)** ADET of leaves in different layers of near-isogenic lines in genetic background of L54 and SZ2. **(I, J)** ABET of leaves in different layers of isogenic lines in genetic background of L54 and SZ2. *p<0.05; **p< 0.01. **(K, L)** PT of leaves in different layers of near-isogenic lines in genetic background of L54 and SZ2. **(M, N)** ST of leaves in different layers of near-isogenic lines in genetic background of L54 and SZ2. **(O, P)** CTR of leaves in different layers of near-isogenic lines in genetic background of L54 and SZ2. **(Q, R)** SR of leaves in different layers of near-isogenic lines in genetic background of L54 and SZ2. *p<0.05; **p< 0.01.

The thickness of sub-okra leaves was lower than that of normal leaves in different layers of the near-isogenic lines in both genetic backgrounds (**Figures 4A–L**). Compared to that of the L54 NORMAL leaves, the LT of L54 SUBOKRA leaves from the top layer to bottom layer was reduced by 5.24%, 11.06%, and 14.80% (**Figure 5A**). In the genetic background of SZ2, the LT of sub-okra leaves from the top layer to the bottom layer was also reduced by 3.73%, 11.72%, and 13.71% (**Figure 5B**). Except for LD, CTR, and SR, the other anatomical parameters of sub-okra leaves were consistently lower than those of normal leaves (**Figures 5C–R**). The LD of sub-okra leaves was consistently increased in different layers of near-isogenic lines in both genetic backgrounds (**Figures 5E, F**). Three morphological parameters exhibited different correlations with other seven anatomical parameters (**Supplementary Figure S6**).

### 3.4 Leaf area index and intercepted PAR rate in the whole growth period

**Figure 6:**
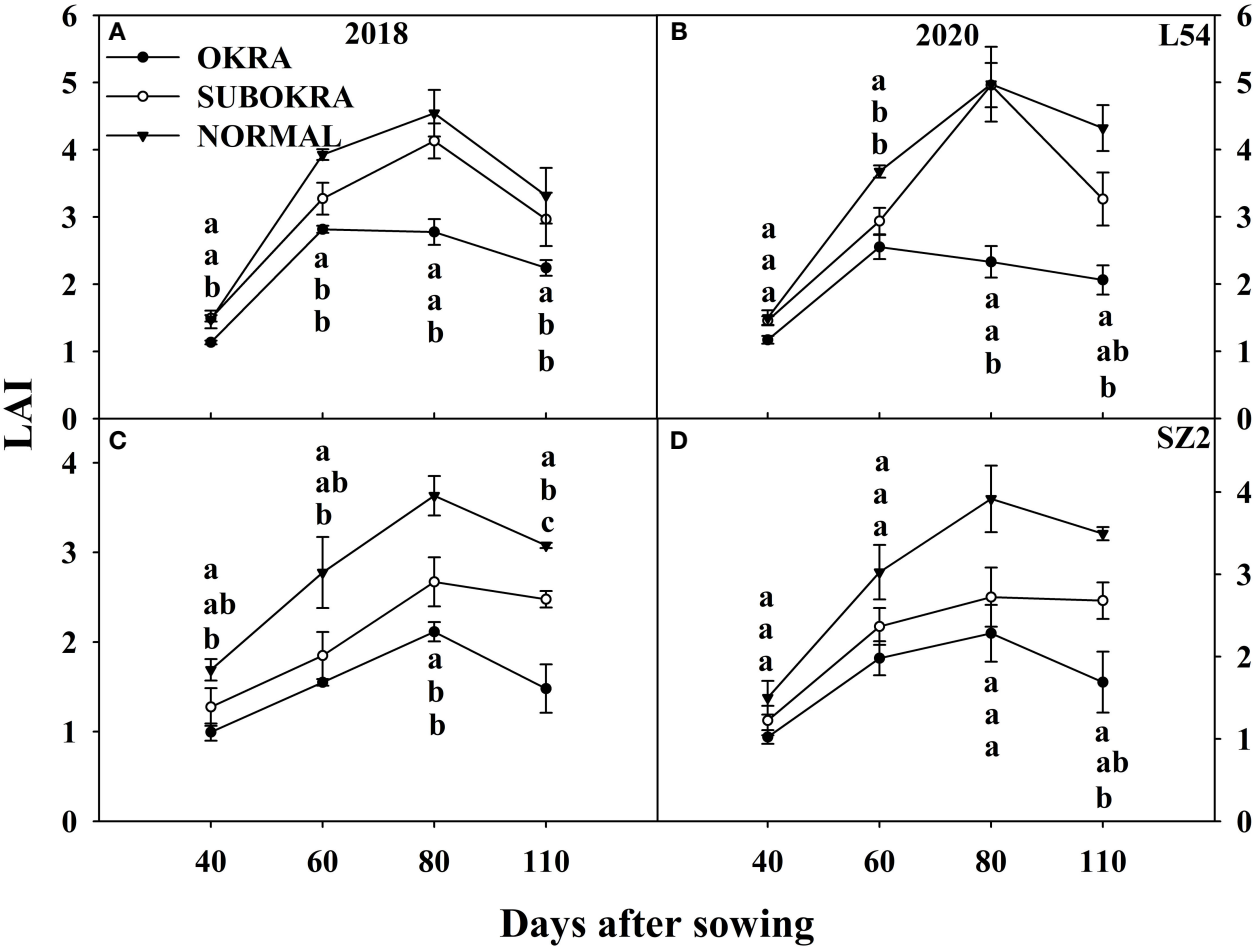
LAI of near-isogenic lines at different stages. **(A, B)** LAI of near-isogenic lines in L54 genetic background in 2018 and 2020. **(C, D)** LAI of near-isogenic lines in SZ2 genetic background in 2018 and 2020. Different lowercase letters are significantly different at p < 0.05.

**Figure 7:**
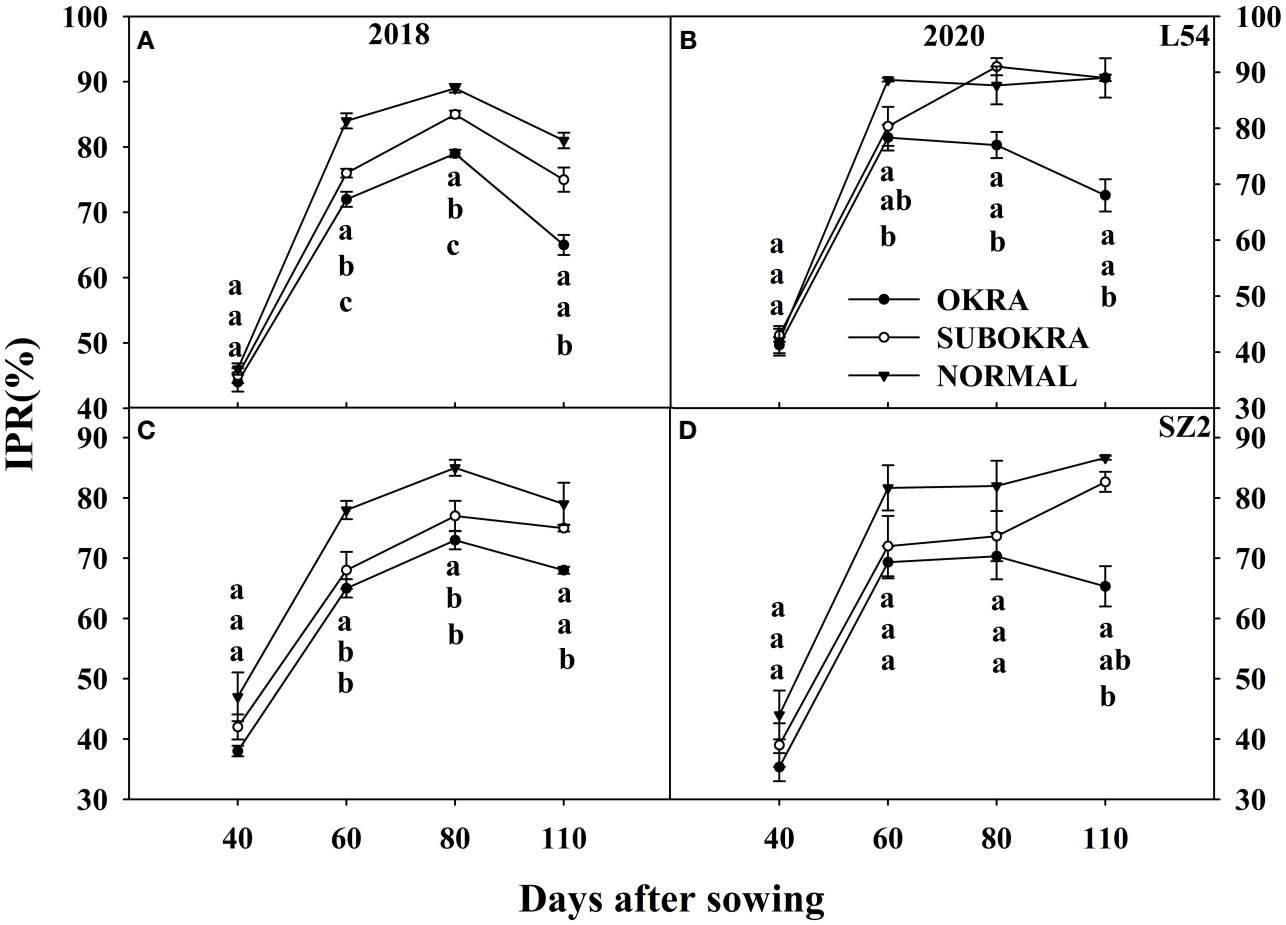
IPR of near-isogenic lines at different stages. **(A, B)** IPR of near-isogenic lines in L54 genetic background in 2018 and 2020. **(C, D)** IPR of near-isogenic lines in SZ2 genetic background in 2018 and 2020. Different lowercase letters are significantly different at p < 0.05.

Modifying the leaf shape directly led to changes in the LAI and IPR (**Figures 6**, **7**). The LAI of lines with sub-okra leaves was higher than that of lines with okra leaves and smaller than that of lines with normal leaves from 40 to 110 DAS, in both genetic backgrounds. The LAI of all lines with sub-okra and normal leaves peaked at 80 DAS. Furthermore, the LAI of L54 SUBOKRA was 0.79%–18.44% lower than that of L54 NORMAL and 21.35%–28.54% smaller than that of SZ2 NORMAL during the growth period.

Similarly, the IPR of the lines with sub-okra and okra leaves was also lower than that of the lines with normal leaves at most growth stages. Compared with that of lines with normal leaves, the IPR of L54 SUBOKRA was reduced by 0.38%–9.46% from 60 to 110 DAS. In the genetic background of SZ2, the IPR of line with sub-okra leaves during the growth period was also reduced by 4.83%–12.32%.

### 3.5 Net photosynthetic rate and chlorophyll content in the whole growth period

**Figure 8:**
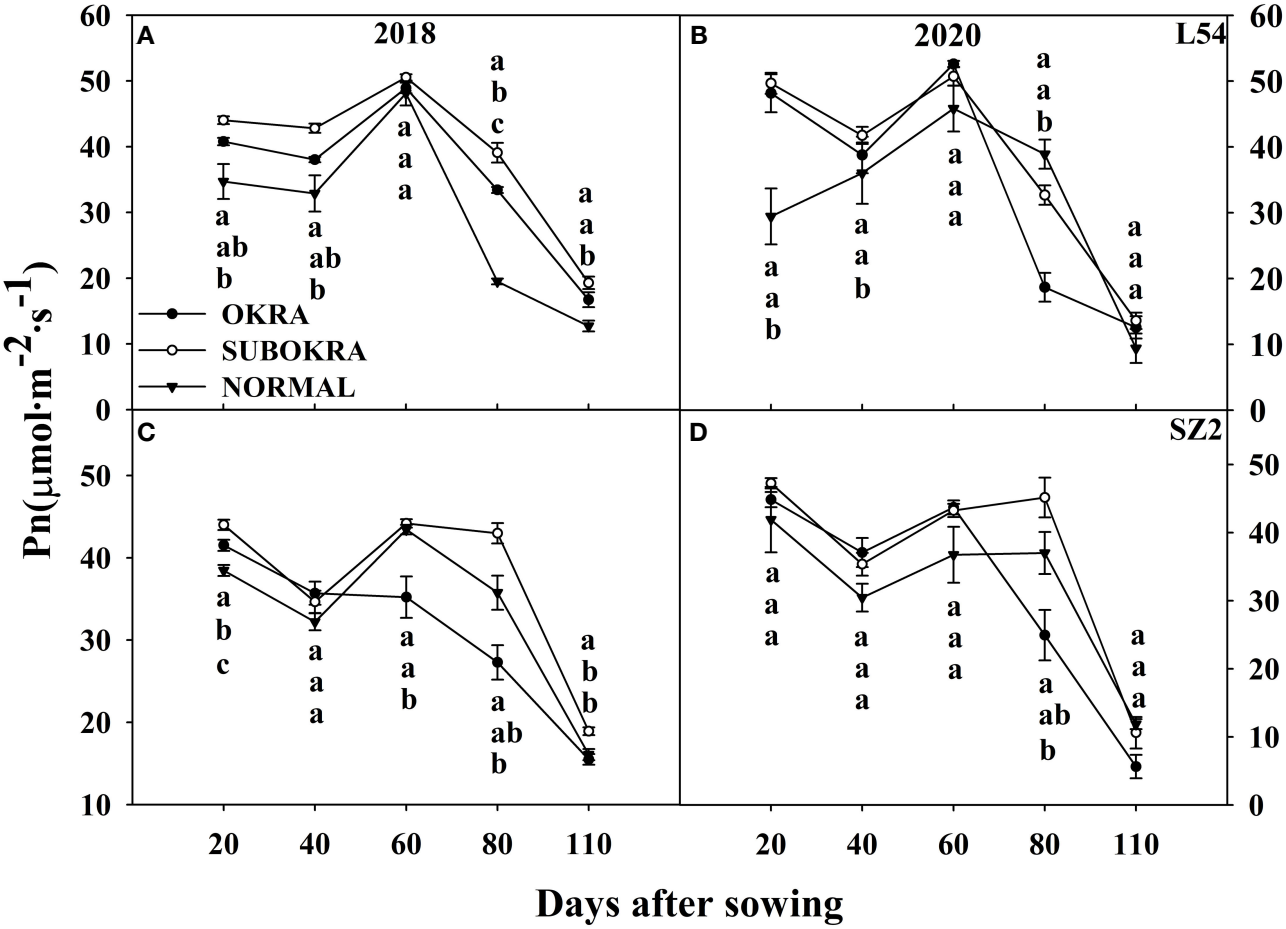
Pn of near-isogenic lines at different stages. **(A, B)** Pn of near-isogenic lines in L54 genetic background in 2018 and 2020. **(C, D)** Pn of near-isogenic lines in SZ2 genetic background in 2018 and 2020. The different lowercase letters are significantly different at p< 0.05.

**Figure 9:**
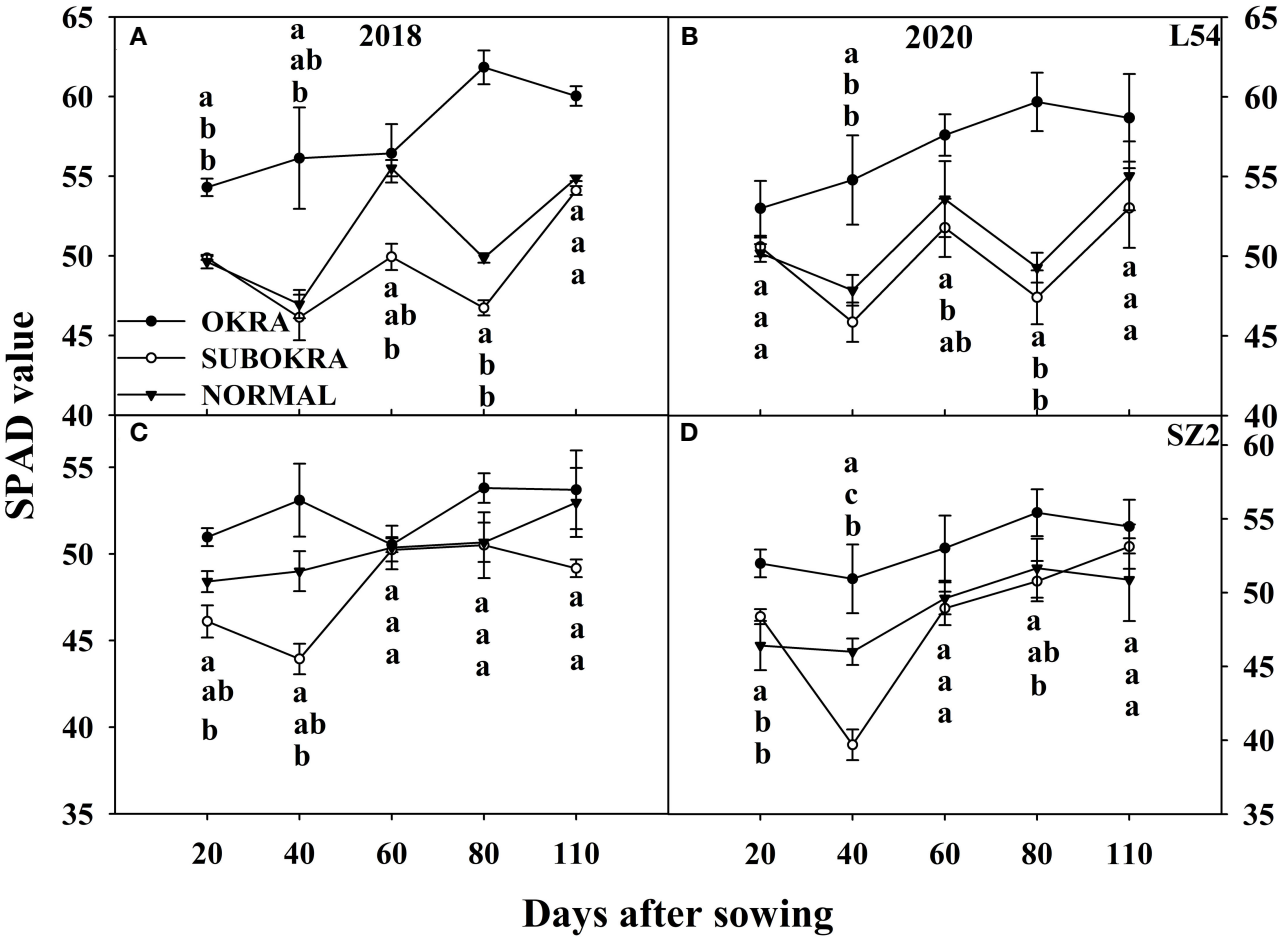
SPAD value of near-isogenic lines at different stages. **(A, B)** SPAD value of near-isogenic lines in L54 genetic background in 2018 and 2020. **(C, D)** SPAD value of near-isogenic lines in SZ2 genetic background in 2018 and 2020. Different low-case letters are significantly different at p < 0.05.

The photosynthetic rates of sub-okra leaves were higher than those of normal and okra leaves at most stages. Specifically, the Pn of L54 SUBORKA was 7.79%–48.56% higher than that of L54 NORMAL during the growth period (**Figures 8A, B**). Compared to the Pn of SZ2 NORMAL, that of SZ2 SUBOKRA increased by 5.65%–21.13% (**Figures 8C, D**). In contrast, the chlorophyll content was lower than those of normal and okra leaves at most stages. Thus, the SPAD values of L54 SUBORKA was 2.54%–6.75% lower than that of L54 NORMAL from 40 to 110 DAS, respectively (**Figures 9A, B**). Consistently, in the SZ2 genetic background, the SPAD values of lines with sub-okra leaves decreased by 0.36%–11.96% during the growth period (**Figures 9C, D**).

### 3.6 Biomass, fiber yield, and quality

**Figure 10:**
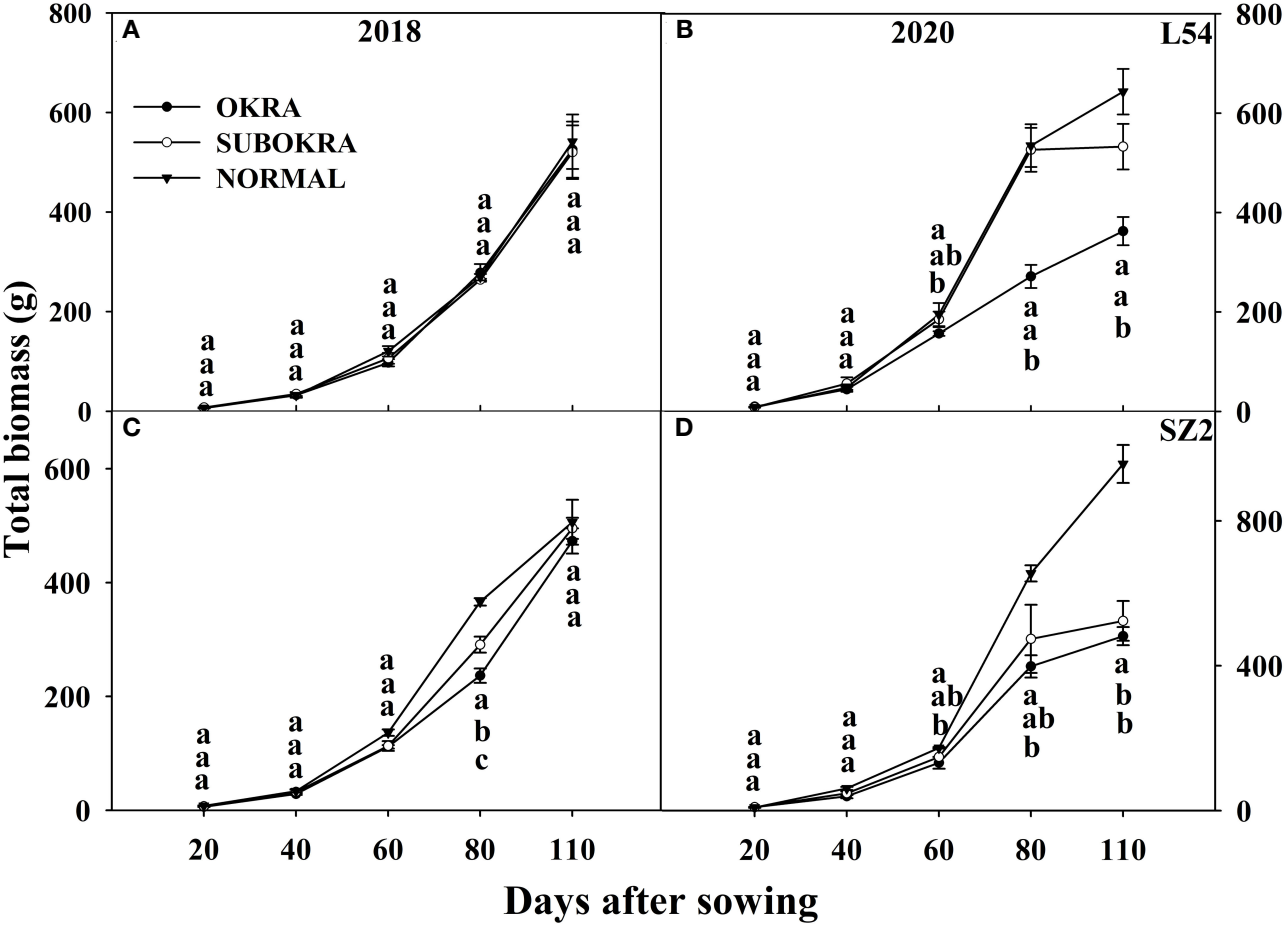
Biomass of near-isogenic lines at different stages. **(A, B)** Biomass of near-isogenic lines in L54 genetic background in 2018 and 2020. **(C, D)** Biomass of near-isogenic lines in SZ2 genetic background in 2018 and 2020. Different lowercase letters are significantly different at p< 0.05.

The total biomass of lines with sub-okra leaves was lower than that of lines with normal leaves and higher than that of lines with okra leaves at most sampling stages. The final biomass of lines with sub-okra leaves increased by 22.93% and 6.72% compared to that of lines with okra leaves in the L54 and SZ2 genetic backgrounds, respectively, and decreased by 10.55% and 23.68% compared to that of lines with normal leaves, respectively (**Figures 10A–D**). Finally, the biomass of each organ in lines with sub-okra leaves was also between that of the other two near-isogenic lines for each genetic background (**Supplementary Figures S7**, **Supplementary Figures S8**).

Overall, the yields of the lines with sub-okra leaves were highest among the three lines for the two genetic backgrounds (**Table 1**). In the L54 genetic background, the first harvest yield and total yield of lines with sub-okra leaves were 8.99% and 8.53% higher than those of lines with normal leaves, respectively. Similarly, the first harvest yield and total yield of lines with sub-okra leaves increased by 3.73% and 2.91%, respectively, compared with those of lines with normal leaves in SZ2 genetic background. Moreover, the sub-okra leaf shape had no negative effect on the lint percentage and fiber quality (**Tables 1**, **2**).

**Table 1:** Effect of leaf shape on yield and lint percentage.

Treatment	Yield of first harvest (kg/ha)	Total yield (kg/ha)	Lint percentage (%)
L54 background			
Leaf shape (LS)			
Okra	2,555.75 b	2,687.54 b	42.04 a
Sub-okra	3,018.46 a	3,378.33 a	40.76 b
Normal	2,769.58 ab	3,112.67 a	41.29 ab
Year(Y)			
Year 2018	3,064.96 a	3,093.62 a	39.72 b
Year 2020	2,497.57 b	3,025.40 a	43.72 a
Source of variance			
Y	1,448,702.70 **	20,940.81 ns	48.38 **
LS	643,533.94 *	1,457,006.34 **	2.47 *
Y×LS	4,807.48 ns	135,196.18 ns	0.80 ns
SZ2 background			
Leaf shape (LS)			
Okra	2,628.71 a	2,761.15 b	36.78 a
Sub-okra	2,715.54 a	2,959.07 a	37.33 a
Normal	2,617.92 a	2,875.32 ab	36.48 a
Year(Y)			
Year 2018	2,436.29 b	2,517.60 b	36.51 a
Year 2020	2,871.82 a	3,212.77 a	37.22 a
Source of variance			
Y	853,606.14 **	2,174,641.22 **	2.25 ns
LS	34,373.75 ns	118,444.44 *	1.11 ns
Y×LS	117,691.89 ns	39,413.04 ns	0.18 ns

Different lowercase letters are significantly different at p < 0.05. *Significant at p < 0.05 and **significant at p < 0.01. ns, non-significant.

**Table 2:** Effect of leaf shape on fiber quality.

Treatment	Fiber length (mm)	Fiber strength(cN/tex)	Micronaire value	Fiber elongation rate	Fiber uniformity index
L54 background					
Leaf shape (LS)					
Okra	28.98 a	29.08 b	5.30 a	7.33 a	84.9 b
Sub-okra	28.91 a	29.58 a	5.10 b	7.28 a	86.55 a
Normal	28.58 a	29.34 ab	5.06 b	7.60 a	85.85 ab
Year(Y)					
Year 2018	28.71 a	29.47 a	5.21 a	7.14 b	85.56 a
Year 2020	28.94 a	29.19 a	5.11 a	7.67 a	85.98 a
Source of variance					
Y	0.2358 ns	0.3335 ns	0.0460 ns	1.2272 **	0.8022 ns
LS	0.2774 ns	0.3756 *	0.0979 **	0.1739 ns	4.1150 ns
Y*LS	0.2234 ns	0.2022 ns	0.0381 ns	0.0072 ns	1.9106 ns
SZ2 background					
Leaf shape (LS)					
Okra	28.96 a	29.47 a	5.00 a	7.28 b	85.40 a
Sub-okra	29.04 a	29.58 a	4.96 a	7.48 a	85.45 a
Normal	29.10 a	29.72 a	4.91 a	7.57 a	85.30 a
Year(Y)					
Year 2018	28.32 b	29.52 a	5.02	6.91 b	85.17 a
Year 2020	29.75 a	29.66 a	4.89	7.63 a	85.60 a
Source of variance					
Y	9.2450 **	0.0868 ns	0.0660 ns	2.3472 **	0.8450 ns
LS	0.0234 ns	0.1004 ns	0.0117 ns	0.5251 *	0.0350 ns
Y×LS	0.2136 ns	0.1060 ns	0.0114 ns	0.0985 ns	0.9817 ns

Different lowercase letters are significantly different at p < 0.05. * Significant at p < 0.05 and **significant at p < 0.01. ns, non-significant.

## 4 Discussion

Sub-okra leaf shape is regulated by *L_2_u* in *Gossypium hirsutum* L. and *L_2_e* in *G. barbadense* L. However, the relationship between *L_2_u* and *L_2_e* is still unclear. After analyzing the sequence of *L_2_* genes from the *Gossypium* genus, the results indicate that the sub-okra leaf is the ancestral leaf shape of tetraploid cotton that gave rise to the okra allele and that the normal leaf shape is a derived mutant allele that came to predominate and define the leaf shape of cultivated cotton (Andres et al., 2017). In the present study, the gene regulating sub-okra leaf shape was identified as *L_2_e* in *G. barbadense* L. (**Figure 1**). The 5′UTR and CDS sequence of this gene were consistent with that of the *L_2_u* reported (Andres et al., 2017; Jiang et al., 2021). This result indicates that *L_2_e* and *L_2_u* may have a common genetic source; however, further evolutionary evidence is needed.

Compared to that of the normal leaf, the decrease in LA of the sub-okra leaf improved the canopy structure and increased the PAR of different canopy layers, indicating that the leaf could maintain higher photosynthesis (**Figures 3A–D**). LMA is a key feature that reflects many essential aspects of leaf economics and can be used to measure the leaf dry-mass investment per unit of light-intercepting leaf area deployed (Wright and Cannon, 2001). LMA is tightly related to thickness of a leaf blade or the density of tissues (Griffith et al., 2016). Species with a low thickness have a higher mesophyll conductance, which limits photosynthetic rate in many species and hence shapes variation in leaf morphology and anatomy (Muir et al., 2013). Compared to normal leaves, sub-okra leaves exhibiting a thinner leaf blade had a lower LMA, which might have contributed to the higher Pn (**Figures 3C, D, G**, **8A–D**).

Leaf photosynthesis is determined by not only biochemical properties but also anatomical features. Lens-shaped epidermal cells can potentially focus light within the upper layers of a leaf, resulting in a high photo flux density, which is called focusing effect (Brodersen and Vogelmann, 2007). A more flattened epidermis could result in a reduced focusing effect (Xiao et al., 2016). Our results showed that the sub-okra leaf had a thinner ADET, which indicated that increased oblateness of the epidermis might relieve the focusing effect in sub-okra leaf (**Figures 5G, H**). The columnar palisade cells can minimize light scattering and enable light to penetrate a leaf, while the spherical spongy cells are more effective in scattering light and thus maximize light absorptance (Brodersen and Vogelmann, 2010; Xiao et al., 2016). In the present study, a decrease in thickness was also detected in both the palisade and spongy mesophyll, which allowed more light to be absorbed by leaves in the lower layers (**Figures 5K–N**).

Changes in canopy architecture that improve photosynthesis may enhance crop productivity (Feng et al., 2012a, Feng et al., 2012b; Huang et al., 2021; Xiao et al., 2021). Furthermore, any variations in leaf shape can significantly modify canopy architecture, which directly affects light interception and solar energy use (Feng et al., 2016). Okra and sub-okra leaf shapes might increase lobe depth and decrease lobe width, thereby changing the LAI and light penetration (Wells et al., 1986). Our previous results showed that okra and sub-okra leaf shapes effectively optimized the canopy structure of full-season cotton (Jiang et al., 2023). The results summarized herein showed that the LAI of lines with sub-okra leaves was higher than that of lines with okra leaves and smaller than that of lines with normal leaves in short-season cotton (**Figure 6**). Concomitant with the decrease in LAI, the IPR was reduced in lines with sub-okra and okra leaf shapes, compared to normal leaves (**Figure 7**). Although maximizing LAI is closely related to a higher yield, a suitable reduction in LAI might equally improve yield by increasing the light energy supply to the lower leaves and canopy radiation use efficiency (Song et al., 2013; Chang et al., 2019). With a modest reduction in LAI, sub-okra leaves improved total biomass and fruit biomass from different genetic backgrounds in full-season cotton (Jiang et al., 2023). Although the yield of lines with the sub-okra leaf shape also increased in short-season cotton, there were differences in biomass accumulation. As for short-season cotton, although the total biomass of line with normal leaf shape was greater than that of lines with okra and sub-okra leaf shape, fruit biomass in lines with sub-okra leaf shape was greater than that in lines with the other two leaf shapes in the normal climate year (**Figures 10A, C**; **Supplementary Figures S6G**, **S7G**). In this study, compared to the normal weather condition of 2018, the increase in precipitation in August and the decrease in sunlight from June to August accounted for the formation of more biomass and ineffective bolls, which increased the biomass of fruit in line with normal leaf shape in 2020 (**Supplementary Figures S1**, **S6G**, **S7G**; **Figure 10B, D**). The results indicated that lines with sub-okra leaf shapes also produced a more effective yield than other lines with okra and normal leaf shapes in short-season cotton.

The Pn reflects the productivity potential of a genotype (Elmore, 1980). A strong relationship between Pn and yield has been demonstrated by elevated [CO_2_] experiments (Bender et al., 1999; Mitchell et al., 1999; Ainsworth et al., 2004). Okra, sub-okra, and similar leaves (normal × okra) exhibited a higher photosynthetic rate than normal leaves (Wells et al., 1986). Semi-okra leaves (normal × super okra) also showed an increased photosynthetic rate at most growth stages (Zhu et al., 2005). In this study, Pn of sub-okra leaves also performed better than that of the other two leaf shapes in two genetic backgrounds of short-season cotton (**Figure 8**). Therefore, we believe that the increase in Pn laid the foundation for yield improvement in lines with sub-okra leaves.

Pn is also determined by other biochemical properties of the leaves (Song et al., 2018). For example, chlorophyll is a key factor in the absorption of light energy. Chlorophyll content is closely related to productivity, which increases with the peak SPAD value (Feng et al., 2016). Our results showed that Pn and productivity of lines with sub-okra leaves increased with a slight decrease in SPAD value (**Figure 9**). Some studies have reported that a decrease in chlorophyll content increased the efficiency of PSII and light incidence on the surface of leaves (Ort et al., 2011; Xiao et al., 2016; Song et al., 2017; Zhou et al., 2023) because the light-harvesting antenna rapidly and reversibly switched into a photoprotected quenched state, in which potentially harmful absorbed energy was dissipated as heat under conditions of excess sunlight (Alexander et al., 2007; Cutolo et al., 2023). The relatively lower chlorophyll content of sub-okra leaves might help alleviate the damage caused to the photosynthetic system by excess high-energy absorption and maintain higher photosynthetic efficiency in short-season cotton. However, this hypothesis must be further verified in future experiments.

Our results showed that sub-okra leaf shape also conferred the same serial advantages, such as decreasing the LMA, LAI, and chlorophyll content, and increasing the Pn in short-season cotton, in which the yield of fiber was improved, but the lint percentage and fiber quality were not affected (**Tables 1**, **2**). The significant yield increase in L54 SUBOKRA indicated that the line with sub-okra leaves showed an even greater advantage on terms of yield in early maturing short-season cotton.

In this study, we mainly focused on the effect of sub-okra leaf shape on canopy and photosynthetic characteristics under a conventional plant density. Plant density is an important factor that regulates canopy light distribution and affects canopy photosynthetic capacity in field-grown cotton (Yao et al., 2016), and it can influence plant size and change leaf azimuthal distributions. The optimum LAI can normally be reached faster in dense plant populations than in sparse ones (Chapepa et al., 2020). Therefore, it is necessary to study the effect of sub-okra leaf shape on canopy structure and photosynthetic capacity under higher plant densities.

## Data availability statement

The raw data supporting the conclusions of this article will be made available by the authors, without undue reservation.

## Author contributions

HJ: Data curation, Formal analysis, Software, Validation, Visualization, Writing – original draft. XM: Conceptualization, Funding acquisition, Writing – review & editing. JS: Methodology, Investigation, Writing – original draft. MG: Methodology, Investigation, Writing – original draft. XZ: Methodology, Investigation, Writing – original draft. CZ: Methodology, Investigation, Writing – original draft. QC: Data curation, Formal analysis, Software, Validation, Visualization, Writing – original draft. YW: Project administration, Resources, Supervision, Writing – original draft. XW: Project administration, Resources, Supervision, Writing – original draft. JW: Project administration, Resources, Supervision, Writing – original draft. YC: Data curation, Formal analysis, Software, Validation, Visualization, Writing – original draft. DZ: Methodology, Investigation, Writing – original draft. FL: Methodology, Investigation, Writing – original draft. WZ: Methodology, Investigation, Writing – original draft. JZ: Conceptualization, Funding acquisition, Writing – review & editing.

## Supplementary material

Supplementary Figure 1 Climate and environmental condition in 2018 and 2020. **(A)** Temperature; **(B)** Precipitation; **(C)** Sunshine hours.

Supplementary Figure 2 Leaf shapes controlled by *L-D_1_* locus in upland cotton. **(A)** Normal leaf shape; **(B)** Okra leaf shape; **(C)** Sub-okra leaf shape; Scale bar, 1cm.

Supplementary Figure 3 Distribution of genomic variations in near-isogenic lines compared to Hai7124. **(A)** L28 NORMAL; **(B)** L28 SUBOKRA.

Supplementary Figure 4 Detection of chromosomal introgression segment with specific DNA markers (M) DNA ladder; (T) TM-1; (N) L28 NORMAL; (H)Hai7124; (S) L28 SUBOKRA;

Supplementary Figure 5 PAR in different canopy layers of near-isogenic lines. **(A, B)** PAR of different layers of near-isogenic lines in genetic background of L54 and SZ2.

Supplementary Figure 6 Correlation network for morphological and anatomical of leaves in different canopy layers of near-isogenic lines. **(A, B)** correlation network of leaves in up layer of lines with sub-okra and normal leaf shape. **(C, D)** correlation network of leaves in middle layer of lines with sub-okra and normal leaf shape. **(E, F)** correlation network of leaves in bottom layer of lines with sub-okra and normal leaf shape. The solid line and dotted line represent positive and negative correlation, respectively. Morphological and anatomical parameters were labeled with ellipse and square, respectively. *, p<0.05; **, p< 0.01.

Supplementary Figure 7 Biomass of different organs of near-isogenic lines at the background of L54 at different stages. **(A, B)** biomass of root of near-isogenic lines at background of L54 in 2018 and 2020; **(C, D)** biomass of stem of near-isogenic lines at background of L54 in 2018 and 2020; **(E, F)** biomass of leaf of near-isogenic lines at background of L54 in 2018 and 2020; **(G, H)** biomass of fruit of near-isogenic lines at background of L54 in 2018 and 2020. Different low-case letters are significantly different at p< 0.05.

Supplementary Figure 8 Biomass of different organs of near-isogenic lines at the background of SZ2 at different stages. **(A, B)** biomass of root of near-isogenic lines at background of SZ2 in 2018 and 2020; **(C, D)** biomass of stem of near-isogenic lines at background of SZ2 in 2018 and 2020; **(E, F)** biomass of leaf of near-isogenic lines at background of SZ2 in 2018 and 2020; **(G, H)** biomass of fruit of near-isogenic lines at background of SZ2 in 2018 and 2020. Different low-case letters are significantly different at p< 0.05.
