# Supplementary figures and images for: Sub-okra leaf shape conferred via chromosomal introgression from Gossypium barbadense L. improves photosynthetic productivity in short-season cotton (Gossypium hirsutum L.)
Source: Front Plant Sci. 2024 Jul 18;15:1393396. doi: 10.3389/fpls.2024.1393396 (PMC11291245; doi:10.3389/fpls.2024.1393396)

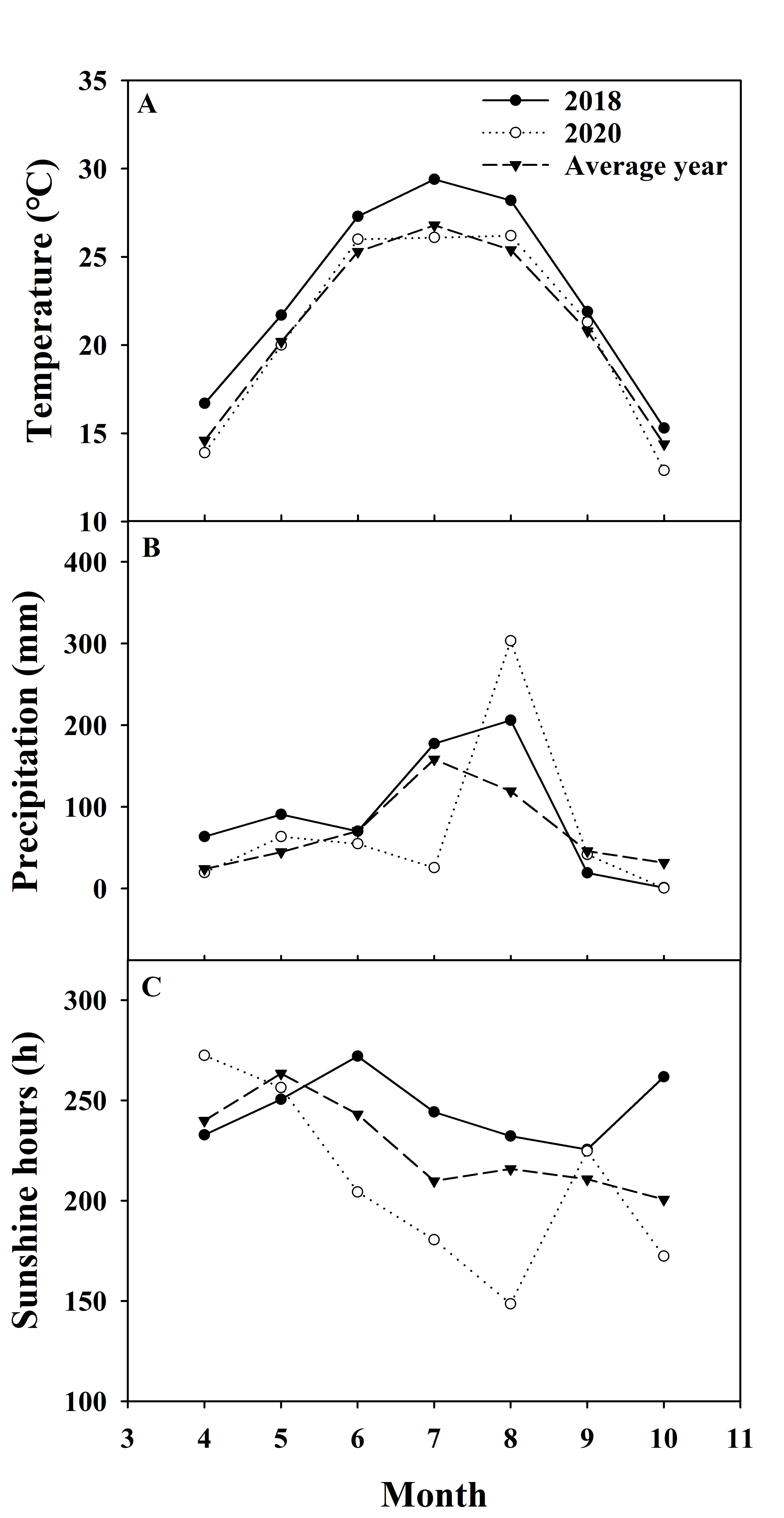

Supplement: Supplementary Figure 1 — Climate and environmental condition in 2018 and 2020. (A) Temperature; (B) Precipitation; (C) Sunshine hours. [file Image_1.jpeg]

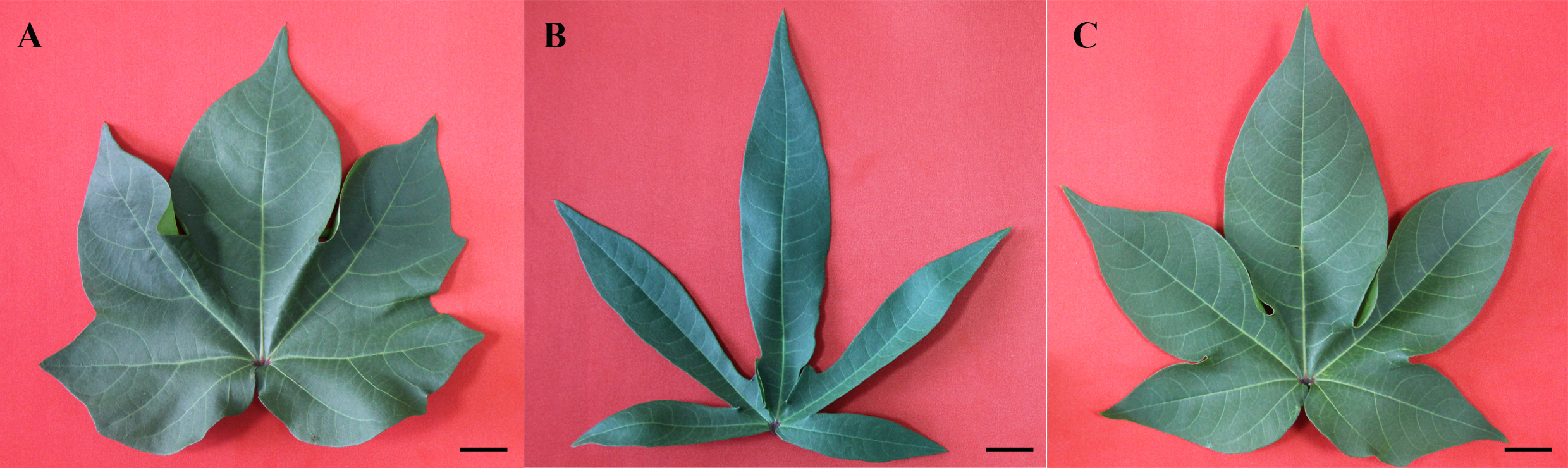

Supplement: Supplementary Figure 2 — Leaf shapes controlled by L-D1 locus in upland cotton. (A) Normal leaf shape; (B) Okra leaf shape; (C) Sub-okra leaf shape; Scale bar, 1cm. [file Image_2.jpeg]

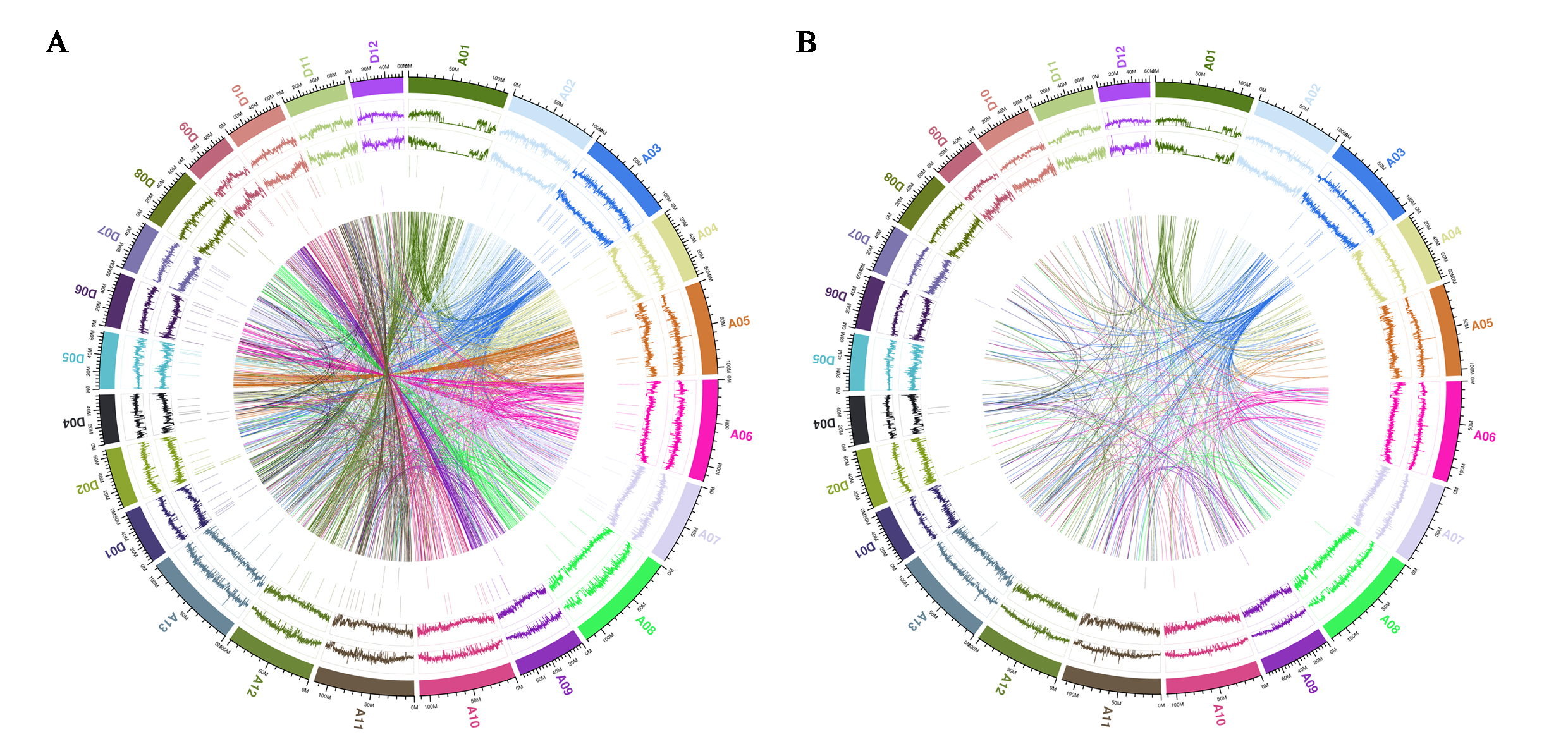

Supplement: Supplementary Figure 3 — Distribution of genomic variations in near-isogenic lines compared to Hai7124. (A) L28 NORMAL; (B) L28 SUBOKRA. [file Image_3.jpeg]

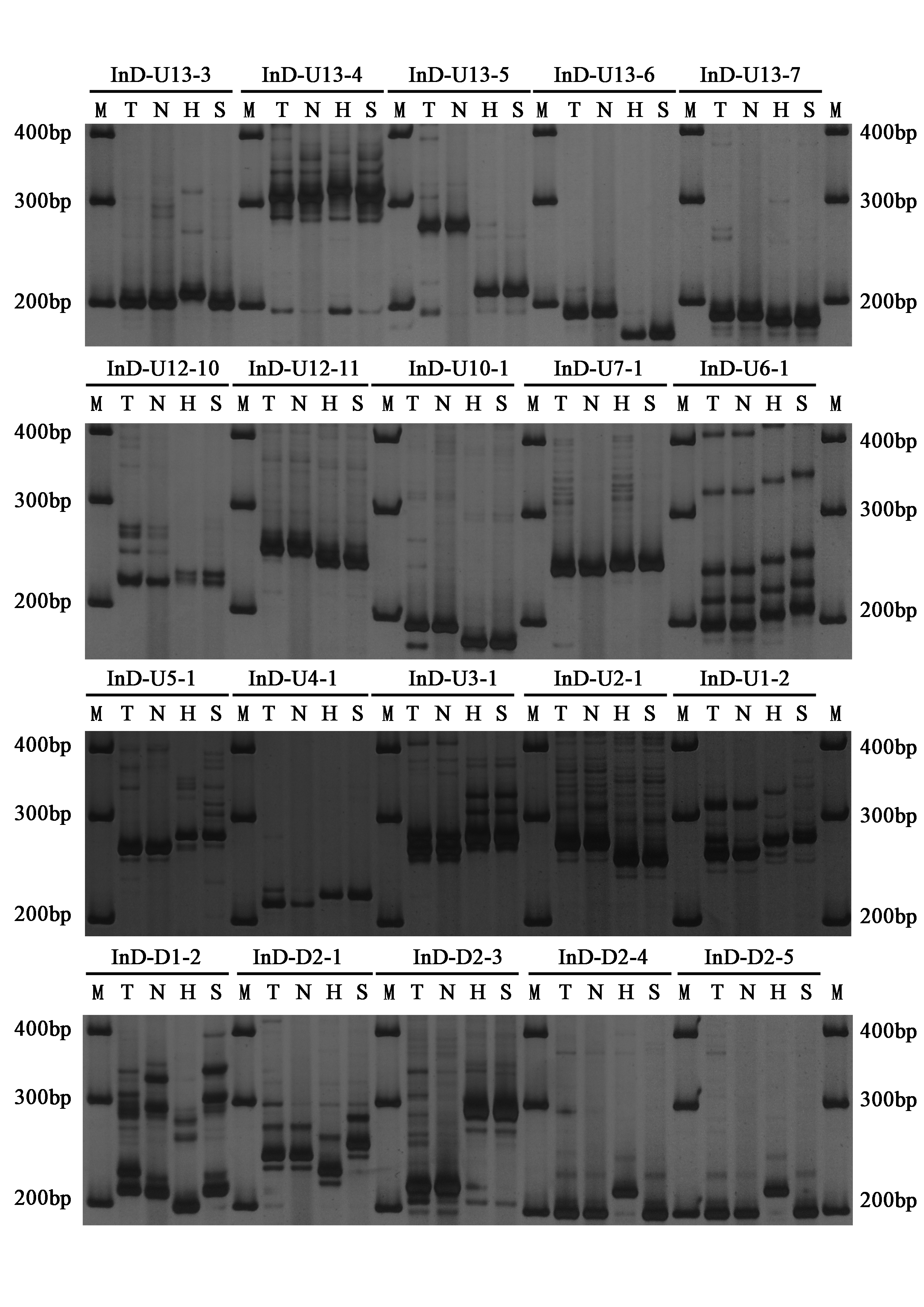

Supplement: Supplementary Figure 4 — Detection of chromosomal introgression segment with specific DNA markers (M) DNA ladder; (T) TM-1; (N) L28 NORMAL; (H)Hai7124; (S) L28 SUBOKRA; [file Image_4.jpeg]

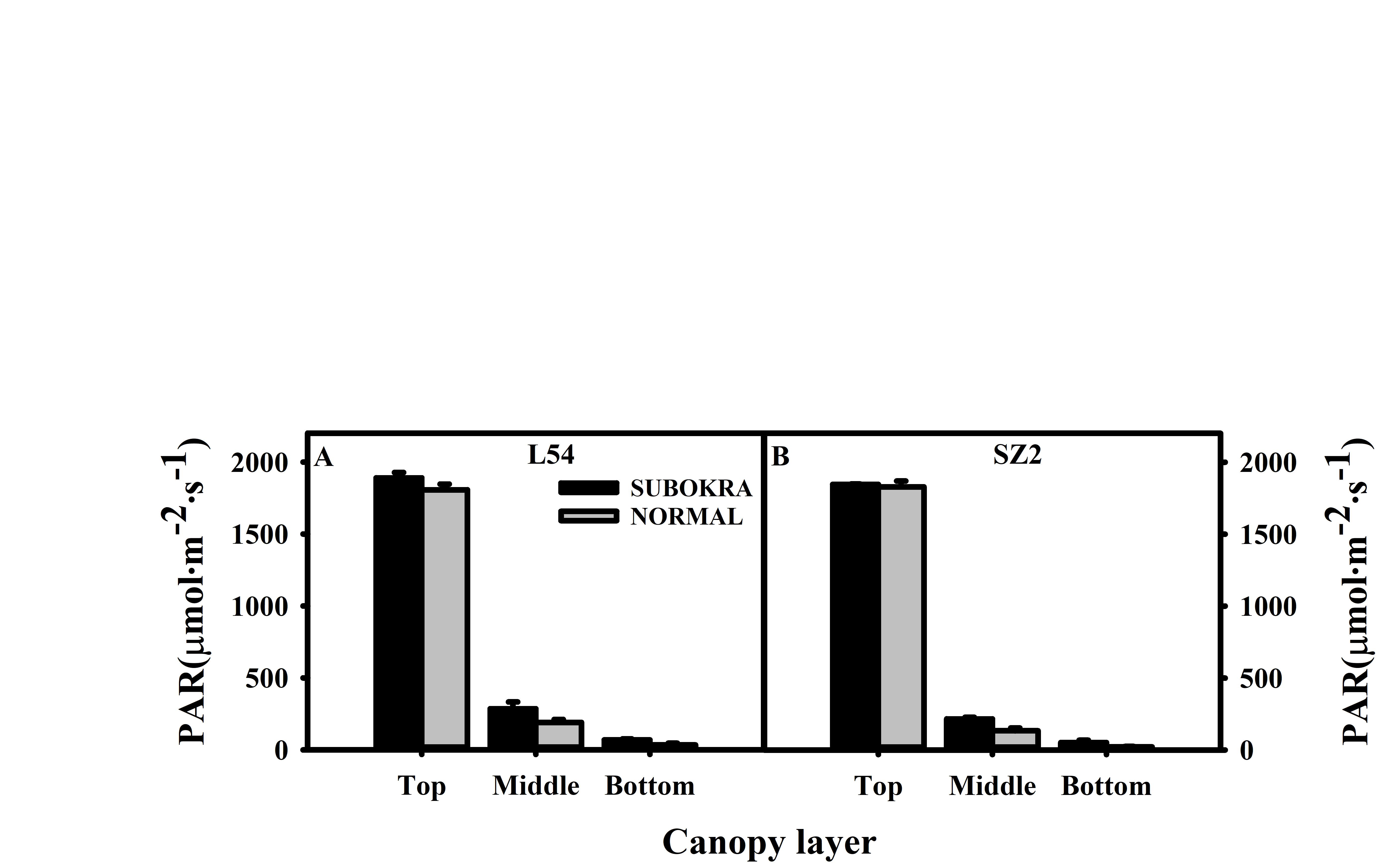

Supplement: Supplementary Figure 5 — PAR in different canopy layers of near-isogenic lines. (A, B) PAR of different layers of near-isogenic lines in genetic background of L54 and SZ2. [file Image_5.jpeg]

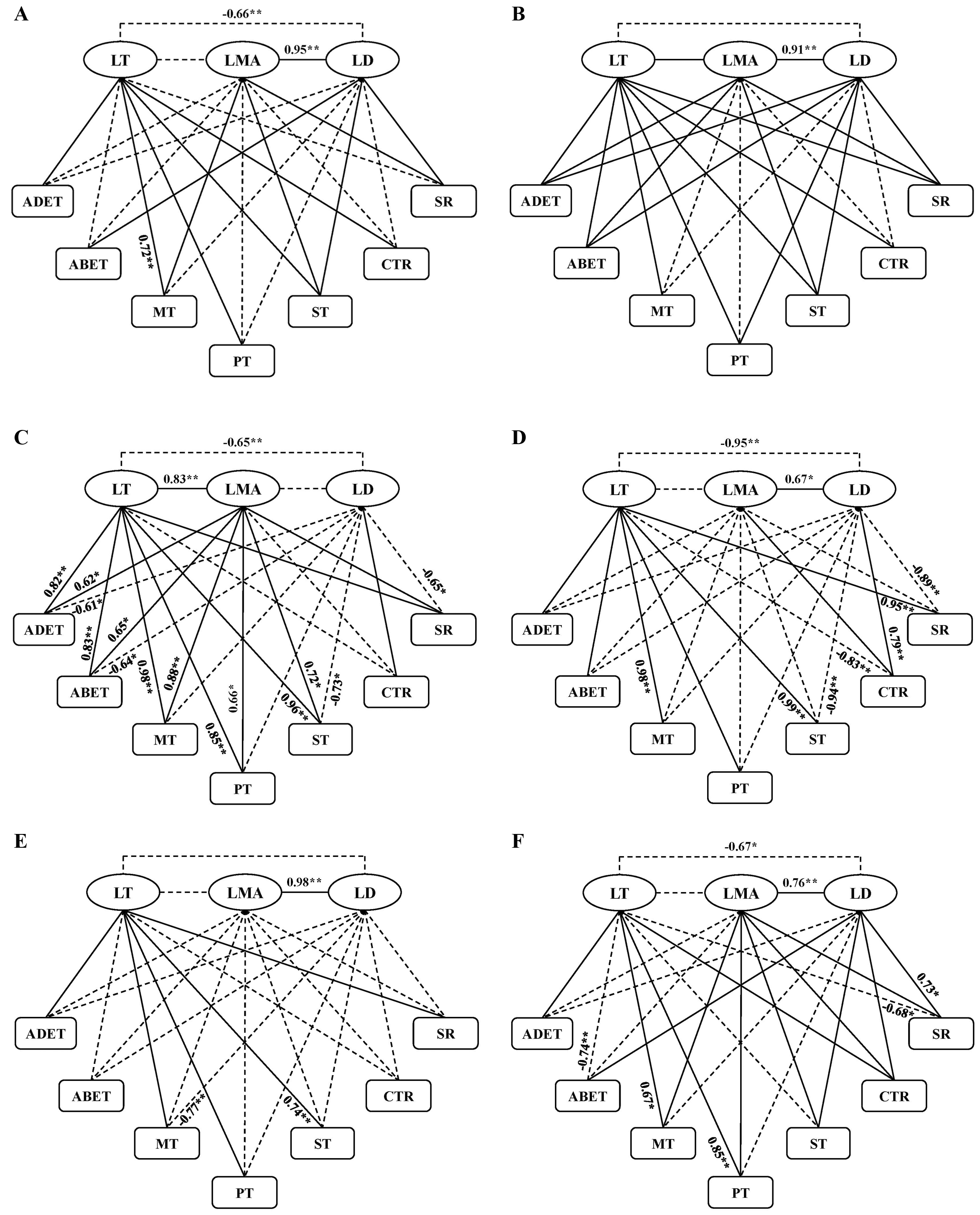

Supplement: Supplementary Figure 6 — Correlation network for morphological and anatomical of leaves in different canopy layers of near-isogenic lines. (A, B) correlation network of leaves in up layer of lines with sub-okra and normal leaf shape. (C, D) correlation network of leaves in middle layer of lines with sub-okra and normal leaf shape. (E, F) correlation network of leaves in bottom layer of lines with sub-okra and normal leaf shape. The solid line and dotted line represent positive and negative correlation, respectively. Morphological and anatomical parameters were labeled with ellipse and square, respectively. *, p<0.05; **, p< 0.01. [file Image_6.jpeg]
